# Blunt splenic injury: Assessment of follow-up CT utility using quantitative volumetry

**DOI:** 10.3389/fradi.2022.941863

**Published:** 2022-07-22

**Authors:** David Dreizin, Theresa Yu, Kaitlynn Motley, Guang Li, Jonathan J. Morrison, Yuanyuan Liang

**Affiliations:** 1 Trauma and Emergency Radiology, Department of Diagnostic Radiology and Nuclear Medicine, School of Medicine, R Adams Cowley Shock Trauma Center, University of Maryland, Baltimore, MD, United States; 2 Department of Diagnostic Radiology and Nuclear Medicine, University of Maryland School of Medicine, Baltimore, MD, United States; 3 Vascular Surgery, R Adams Cowley Shock Trauma Center, University of Maryland School of Medicine, Baltimore, MD, United States; 4 Epidemiology and Public Health, University of Maryland School of Medicine, Baltimore, MD, United States

**Keywords:** quantitative CT, volumetry, follow-up, blunt splenic injury (BSI), computed tomography, spleen, trauma, abdominal trauma

## Abstract

**Purpose:**

Trials of non-operative management (NOM) have become the standard of care for blunt splenic injury (BSI) in hemodynamically stable patients. However, there is a lack of consensus regarding the utility of follow-up CT exams and relevant CT features. The purpose of this study is to determine imaging predictors of splenectomy on follow-up CT using quantitative volumetric measurements.

**Methods:**

Adult patients who underwent a trial of non-operative management (NOM) with follow-up CT performed for BSI between 2017 and 2019 were included (*n* = 51). Six patients (12% of cohort) underwent splenectomy; 45 underwent successful splenic salvage. Voxelwise measurements of splenic laceration, hemoperitoneum, and subcapsular hematoma were derived from portal venous phase images of admission and follow-up scans using 3D slicer. Presence/absence of pseudoaneurysm on admission and follow-up CT was assessed using arterial phase images. Multivariable logistic regression was used to determine independent predictors of decision to perform splenectomy.

**Results:**

Factors significantly associated with splenectomy in bivariate analysis incorporated in multivariate logistic regression included final hemoperitoneum volume (*p* = 0.003), final subcapsular hematoma volume (*p* = 0.001), change in subcapsular hematoma volume between scans (*p* = 0.09) and new/persistent pseudoaneurysm (*p* = 0.003). Independent predictors of splenectomy in the logistic regression were final hemoperitoneum volume (unit OR = 1.43 for each 100 mL change; 95% CI: 0.99–2.06) and new/persistent pseudoaneurysm (OR = 160.3; 95% CI: 0.91–28315.3). The AUC of the model incorporating both variables was significantly higher than AAST grading (0.91 vs. 0.59, *p* = 0.025). Mean combined effective dose for admission and follow up CT scans was 37.4 mSv.

**Conclusion:**

Follow-up CT provides clinically valuable information regarding the decision to perform splenectomy in BSI patients managed non-operatively. Hemoperitoneum volume and new or persistent pseudoaneurysm at follow-up are independent predictors of splenectomy.

## Introduction

The spleen is the most commonly injured organ in blunt abdominal injury (1, 2). The spleen is involved in innate and adaptive immunity and response to bacterial infection (3). Splenectomy carries an estimated 5% lifetime risk of overwhelming post-splenectomy infection (OPSI)- a form of fulminant sepsis associated with a mortality between 50 and 80% (4–6). The prevailing treatment strategy for blunt splenic injury (BSI) has moved away from routine operative management to one of selective non-operative management (NOM), involving attempts at salvaging splenic function in hemodynamically stable patients (1, 5, 7). Trials of NOM, with or without adjunct splenic embolization, also allow patients to potentially avoid unnecessary short-term morbidity associated with laparotomy (8). An examination of the American College of Surgeons National Trauma Data Bank found that ∼85–90% of patients with blunt splenic trauma do not require urgent splenectomy and may be candidates for NOM (9). The failure rate of splenic salvage in patients selected for NOM has a relatively low reported incidence of 8–11% (10–12) and most failures occur within the first 24 h (11).

Failure of non-operative management carries its own increased risk of morbidity and mortality (11, 13) and requires vigilant patient monitoring. There has been variable adoption of follow-up CT in splenic NOM protocols, and this practice remains the subject of debate, with no established consensus at the present time (9, 14–22).

American Association for the Surgery of Trauma (AAST) splenic injury grades correlate with the risk of NOM failure after BSI. In a 2012 systematic review of the literature and meta-analysis of 25 studies Banghu et al. found that AAST grade IV-V injuries and moderate to large hemoperitoneum were significantly associated with increased risk of non-operative management failure (23). In a more recent prospective study of 87 patients in whom AAST grades were assigned based on the patient’s admission CT scan, Brillantino et al. found no significant difference in the success rate of NOM based on AAST scoring (24).

While initial management is determined by a combination of the patient’s hemodynamic status and admission CT imaging (20), the perceived value of follow-up inpatient CT imaging in patients selected for NOM lies in the ability to track the natural history of splenic injury and directly visualize potential leading indicators of delayed splenic rupture. These include worsening hemoperitoneum, laceration, subcapsular hematoma, and new or non-resolving pseudoaneurysms. The use of follow-up CT alongside vital sign monitoring and serial hemoglobin testing during inpatient NOM trials has been found to have utility in several published studies (14, 17, 18).

Cumulative radiation from repeat scanning remains a concern in BSI patients (25) and must be weighed when considering whether to adopt an NOM protocol incorporating follow-up CT. Given conflicting findings, ongoing debate, the scarcity of literature on the topic, and radiation concerns, the role of follow-up CT remains controversial. According to a 2019 international survey of practice patterns, follow-up CT is used for BSI patients selected for NOM in 38% of trauma centers (26). More study is needed to clarify the role of follow-up imaging and the individual signs predictive of splenectomy.

Where employed, follow-up CT is typically performed within 24–72 h (15, 16, 20, 26). Arterial phase CT images are superior for assessment of the presence or absence of pseudoaneurysm (27, 28), while splenic laceration, and subcapsular hematoma are graded in a coarse categorical fashion per AAST grading criteria on portal venous phase images where peak parenchymal enhancement helps delineate organs from adjacent non-enhanced blood (29). Hemoperitoneum volume assessment is usually performed using a coarse subjective method described by Federle and Jeffrey (30), although recently, voxelwise quantitative CT measurement of hemoperitoneum volume has been shown to be more predictive of outcomes in trauma patients than the Federle method (31). Even though hemoperitoneum and other bleeding features in the abdominopelvic region are typically irregular, and multifocal, volumetric measurements have been shown to have high interobserver agreement and repeatability (31–34). In a previous study on hemoperitoneum segmentation, test-retest reliability of repeat hemoperitoneum measurements was high, with both interclass correlation coefficient and Pearson *r* values of 0.98 (31). CT volumetry provides granular measurements and has been leveraged to study tumor growth rate as a marker of outcome on repeat CT imaging (35, 36) but this is not well-explored in the trauma domain.

The purpose of this study is to model features of BSI in NOM patients followed with surveillance CT as predictors of splenectomy. Voxelwise measurements of features scored categorically using AAST and Federle grading are measured using CT volumetry to provide a greater level of granularity. The performance of our model is compared with admission CT-based AAST grading as a benchmark.

## Materials and methods

### Institutional management approach

At our trauma center, in light of the immunological consequences of a potentially avoidable splenectomy, we take a conservative approach using a standard surveillance policy incorporating follow-up contrast-enhanced CT (CECT). Patients sufficiently stable to undergo an initial admission CECT are graded per AAST criteria. Those with grade 5 AAST injuries including active hemorrhage undergo laparotomy.

### Follow-up CT in NOM without angiography

Stable patients with AAST grade 2 injuries and no concurrent visceral injuries necessitating laparotomy are triaged for follow-up CT imaging, which typically occurs between 24 and 48 h.

### Follow-up CT in NOM with angiography

Those with AAST grade 3 and 4 injuries undergo routine interrogation with angiography and possible angioembolization prior to follow-up CT. Follow-up CT after angiography is typically performed between 48 and 72 h (37). Patients with pseudoaneurysm associated with minimal parenchymal disruption may be observed on a case-by-case basis. All NOM patients with or without angiography are also followed with serial CBC and abdominal exams.

### Splenectomy or splenic salvage after follow-up CT

The decision to perform splenectomy is ultimately based on individual surgeon discretion, however it is informed by varying combinations of worsening on follow-up CT (19), deteriorating hemodynamic status (38), or ongoing need for transfusion (39). Worsening on follow-up CT is considered indicative of splenic rupture or high risk thereof. It is characterized by increasing splenic parenchymal disruption, subcapsular hematoma, or hemoperitoneum as well as new or persistent pseudoaneurysm (14, 17, 18). Our protocol does not involve repeat attempts at angiography after follow-up CT. To avoid the potential catastrophic consequences of a delayed splenic rupture, where the surgent deems there is sufficient injury progression, splenectomy is offered.

### Patient selection

This work was part of an institutional review board-approved and HIPAA-compliant study performed at University of Maryland Medical Center and included a retrospectively analyzed cohort of consecutive adult (age ≥ 18) patients identified using our electronic medical record who underwent an inpatient trial of NOM with follow-up arterial and portal venous phase contrast-enhanced CT through the abdomen and pelvis between July 1, 2017, and June 30, 2019, with follow-up of splenic injury as the primary indication. Patients who underwent urgent laparotomy following admission (*n* = 11), or who had follow-up CT for indications other than splenic trauma (*n* = 9) were excluded. The final study cohort was composed of 51 patients (median age 40; 59% male). Splenectomy served as the primary endpoint (*n* = 6). 45 patients underwent successful splenic salvage (*n* = 45).

Demographic information and results of clinical and laboratory tests were extracted from the electronic medical record (Table 1) including: patient age; gender; systolic blood pressure (SBP), heart rate (HR), hemoglobin (Hgb), and number of units of packed red blood cells transfused (PRBCs) at the time of follow-up imaging. The shock index was derived from HR and SBP. Other covariates collected included the AAST splenic organ injury scale (OIS) grade on admission CT; whether NOM involved proximal or distal adjunct splenic artery embolization; whether angioembolization led to successful splenic salvage or was followed by splenectomy; and days between initial and follow-up imaging (approximated to the nearest minute using study time stamps).

### Image analysis

Dual arterial and portal venous contrast-enhanced abdominopelvic trauma CTs were performed from the dome of the diaphragm to the greater trochanters with one of two trauma bay scanners- either a dual source 128-section CT (SOMATOM Force; Siemens, Erlangen Germany), or a 64-section CT (Brilliance; Philips Healthcare, Andover, Mass.) Additional scan parameters included the use of 100 mL of 350 mg/mL Iohexol (Omnipaque; GE healthcare; Boston, Mass.), bolus tracking in the descending aorta individualized by scanner for arterial phase scan timing, followed by a 60–70 s delay for the portal venous phase. Images were archived at 1.5 mm section thickness.

Voxelwise measurements of splenic laceration, hemoperitoneum, and subcapsular hematoma were derived from portal venous phase (PVP) images of the initial and follow-up scans in 3D Slicer (version 4.10.2) (40) labeled in three planes using the 3D threshold paint tool set to ∼30–80 HU, following methodology described in Dreizin et al. (31, 41, 42). Labeling was performed by a trained research assistant and all scans were subsequently reviewed and edited by a trauma radiologist attending with 10 years of experience. Following manual segmentation, total volumes of each imaging feature were automatically calculated and recorded in milliliters (mL). Presence or absence of new or persistent pseudoaneurysm on follow-up CT was assessed using arterial images.

### Statistical analysis

Stata/SE (version 17; College Station, TX) was used for all statistical analysis. For continuous variables, the Mann-Whitney *U* test was used to compare non-normally distributed data between the splenectomy and splenic salvage groups while a *t*-test was used to compare mean values of normally distributed data between the two groups. Fisher’s exact test was used to compare proportions between the splenectomy and splenic salvage groups.

Multivariable logistic regression was used to construct a model including imaging predictors at a significance level of 10% in bivariate analysis (i.e., *p* < 0.10). Variables included in the full model included final hemoperitoneum volume; final subcapsular hematoma volume; change in subcapsular hematoma volume per day; and presence or absence of pseudoaneurysm on follow-up CT. A backward model selection procedure was used to identify a final reduced model with all predictors that were statistically significant at a level of 10%.

The Hosmer-Lemeshow goodness-of-fit test with 10 quantiles of estimated probabilities was used to determine whether model-predicted probabilities conformed to the observed data, with a *p* > 0.05 indicating goodness of fit. Area under the ROC curve (AUC) was calculated for the model and assessed qualitatively as a measure of accuracy using a commonly employed grading scale: 0.5–0.59, fail; 0.6–0.69, poor; 0.7–0.79, fair; 0.8–0.89, good; AUC 0.9–1, excellent (43). The model AUC was compared with the AUC of AAST grading.

## Results

### Baseline characteristics

NOM with follow-up CT was attempted in 51 patients, with 6 patients (12% of the cohort) ultimately requiring splenectomy. Baseline demographic and clinical characteristics for the splenectomy and splenic salvage groups are shown in Table 1. A median of 2.0 days transpired between admission and follow-up CT (IQR: 1.3–2.6 days). There was a trend toward less time between CTs in the splenectomy group indicating a greater index of clinical suspicion for delayed splenic rupture (DSR), however this did not reach statistical significance (*p* = 0.11). There were no significant differences in age (*p* = 0.56), gender (*p* = 0.68), SBP (0.41), HR (0.73), shock index (*p* = 0.77), and change in Hgb (*p* = 0.11) between the splenectomy and splenic salvage groups, however this may have been related to more aggressive transfusion in the splenectomy group. A mean of 3.0 units PRBCs were transfused in the splenectomy group vs. 1.5 units in the splenic salvage group (*p* = 0.15). Three patients (one of whom underwent proximal AE) were offered splenectomy after the second study due to perceived high risk of DSR, and 3 patients progressed to DSR on the follow-up exam.

In total, 10 patients underwent AE, with proximal splenic artery embolization in all 10 patients. One patient in the splenic salvage group had a combined proximal and selective coil embolization. There were no significant differences in AAST grade distributions between the splenectomy and splenic salvage groups (*p* = 0.63).

### Predictors (bivariate analysis)—pseudoaneurysm

In total 13 patients (25%) had pseudoaneurysm and7 of these patients underwent AE. The proportion of patients with new or persistent pseudoaneurysm on follow-up CT was significantly higher in patients who required splenectomy after failing a trial of NOM (*p* = 0.003). Pseudoaneurysm on follow-up CT was present in 83% (5/6) of patients who underwent splenectomy and in 18% (8/45) of patients with successful splenic salvage. One patient failed AE and underwent splenectomy for splenic laceration and massive hemoperitoneum (1,415 mL), despite non-visualization of pseudoaneurysm on the follow-up CT scan.

### Predictors (bivariate analysis)—volumetric measurements

Table 2 details the final volumes of hemoperitoneum, laceration, and subcapsular hematoma on follow-up CT as well as the rate of change. Only final hemoperitoneum volume (*p* = 0.003), final subcapsular hematoma volume (*p* = 0.001) and change in subcapsular hematoma volume per day (*p* = 0.09) met significance for inclusion in logistic regression, with higher values in the splenectomy group. Final laceration volume and the rate of change in volume for hemoperitoneum, and laceration did not vary significantly between groups.

### Multivariable logistic regression model

Predictor variables that remained in the final model after backward elimination steps included hemoperitoneum volume (OR = 1.43 per 100 mL change) and new/persistent pseudoaneurysm (OR = 160.3). Results of regression are shown in Table 3.

**FIGURE 1:**
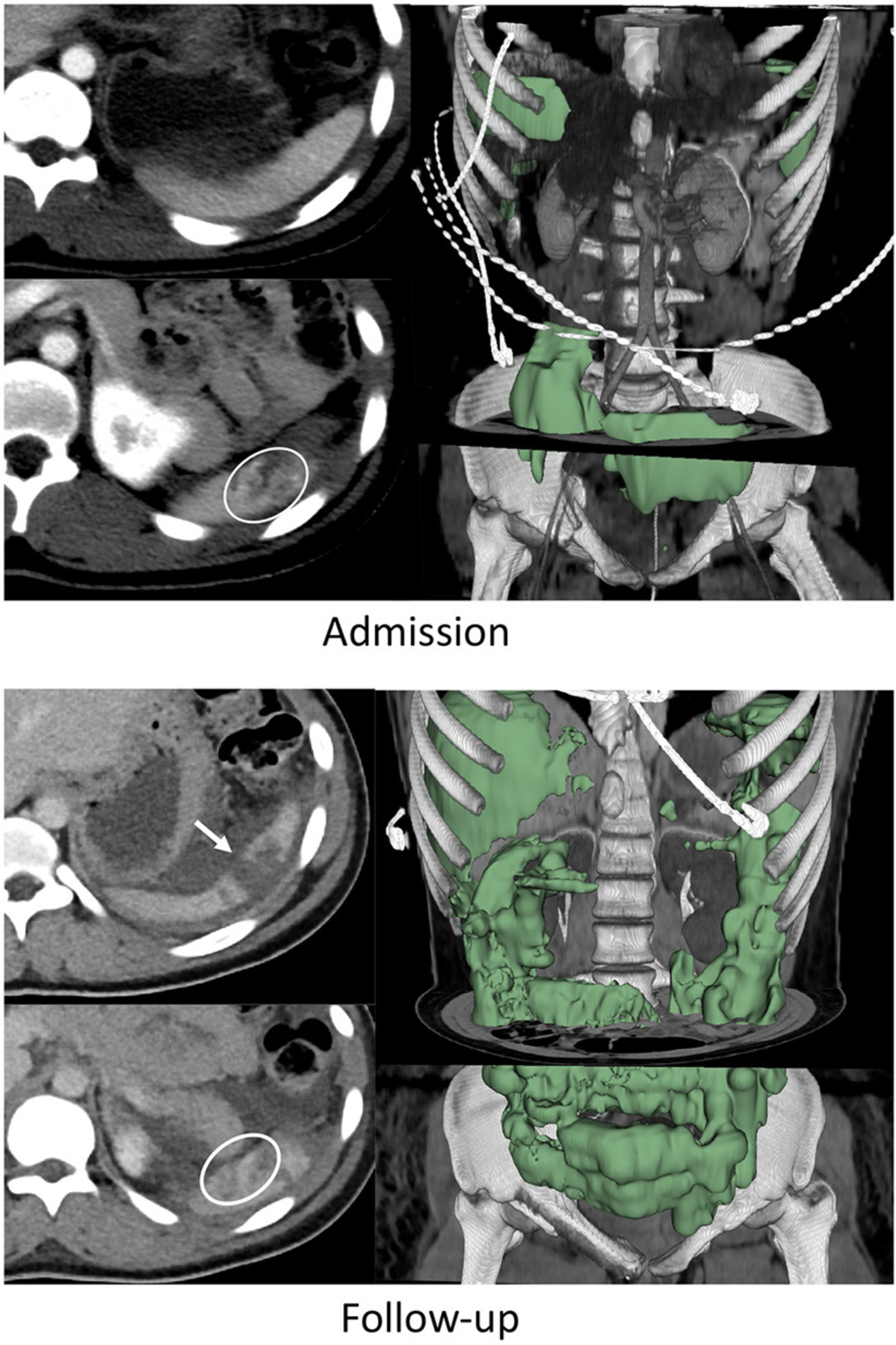
Nineteen years-old female with BSI who failed AE and underwent splenectomy. On admission CT, the patient had a grade IV BSI with several pseudoaneurysms in the inferior pole of the spleen (circle on bottom left admission CT image), and 253 mL of hemoperitoneum. This increased to 1,415 mL on follow-up CT performed 49 h later (green label mask, right image). Pseudoaneurysms were no longer visualized but the laceration had expanded to include the upper pole (arrow on top left follow up image).

**FIGURE 2:**
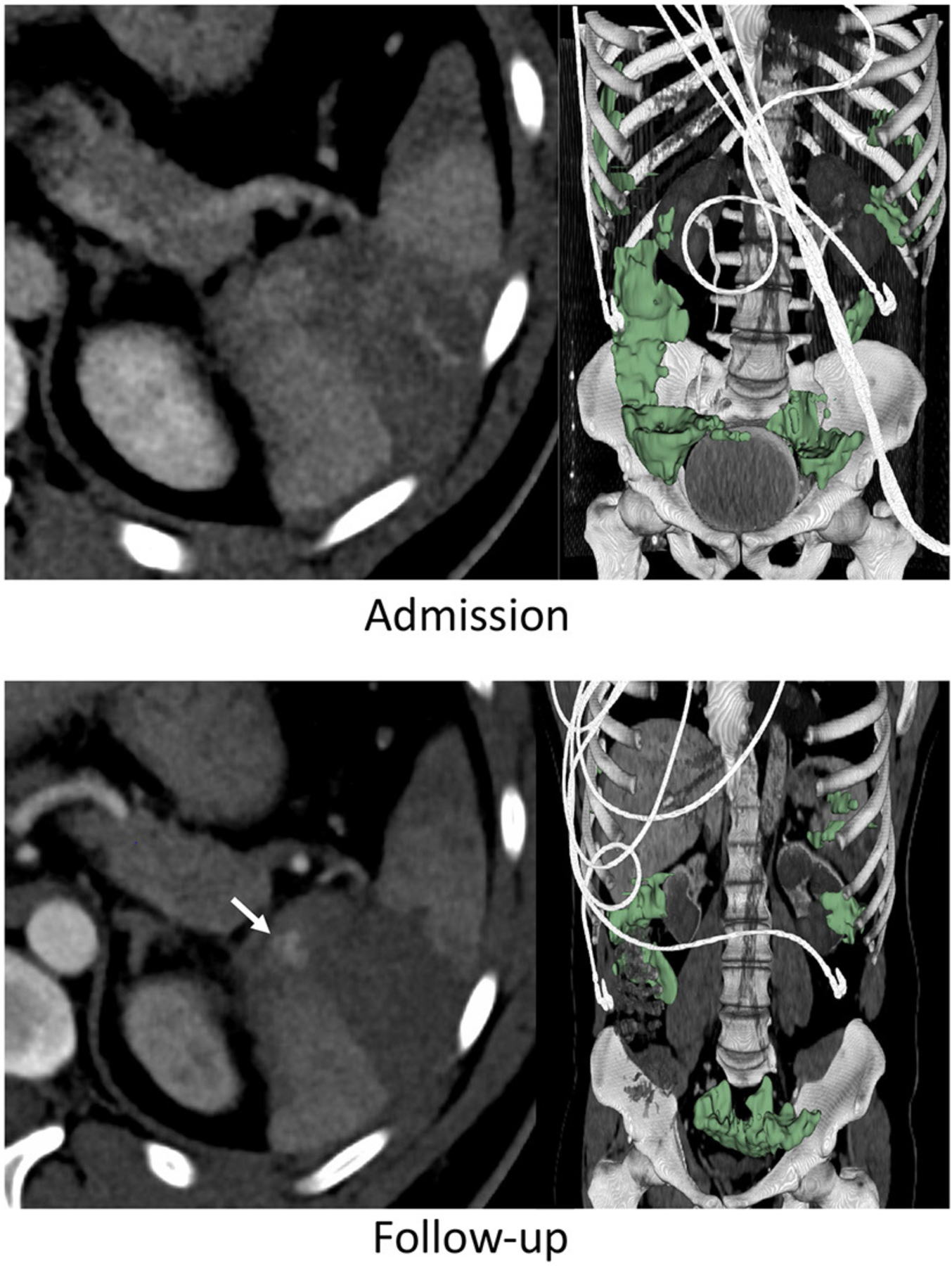
Thirty three years-old male with BSI successfully managed non-operatively with adjunct AE. Admission CT shows an AAST grade 3 injury. A new pseudoaneurysm was seen on follow-up performed 25 h later (arrow) but hemoperitoneum volume (green label mask) had decreased from to 262 to 222 mL.

AUC for the model was in the “excellent” range at 0.91 (95% CI: 0.82–0.99). Hosmer-Lemeshow goodness of fit test produced a *p*-value of 0.98, indicating that the model fits the observed data. The AUC for the model was significantly higher than the AUC for admission AAST grading of 0.59 (95% CI: 0.37–0.82, *p* = 0.025). Examples of initial and follow-up CT imaging for patients who underwent either splenectomy or successful splenic salvage are provided in Figures 1, 2.

### Radiation dose

The mean effective dose was 21.3 millisieverts (mSv) (95% CI: 18.2–24.4) for the first scan, and 16.1 mSv (95% CI: 13.1–19.1) for the follow-up scan. On average, the effective dose was 5.2 mSv less on the follow up than on the initial admission exam, with a combined dose of 37.4 mSv (95% CI: 32.4–42.4).

## Discussion

Trials of non-operative management have become the standard of care for BSI in initially hemodynamically stable patients. The decision to operate is based on data that suggests a high risk of delayed splenic rupture, and splenectomy is performed in an effort to reduce the risk of uncontrolled splenic bleeding. Vital signs, hemoglobin levels, abdominal signs and symptoms, and blood transfusion requirements are important factors in operative decision-making in patients initially selected for NOM (22, 44). To date, there is a lack of consensus regarding whether follow-up CT has added utility in determining the need for splenectomy after BSI and if so, which CT features are most predictive (14, 15, 17–19, 21, 45). While these issues remain understudied, up to 38% of trauma centers have adopted routine follow up screening CTs in their NOM protocols for BSI patients as of 2019 (26).

Previous reports examining the role of follow-up CT determined that the presence of pseudoaneurysm (19) and subjective hemoperitoneum grading (18) are important predictors of non-operative failure. Multivariable modeling using voxelwise CT measurements of features typically graded in a subjective categorical fashion have been employed in this work to add a greater degree of granularity and objectivity.

We developed a logistic regression model that predicts non-operative failure in patients initially selected for NOM and rescanned with CT during their hospital course with an AUC of 0.91. Only final hemoperitoneum volume and new or persistent pseudoaneurysm on follow-up CT were predictive of NOM failure and splenectomy in multivariable analysis. Pseudoaneurysm predicted splenectomy with an odds ratio of 160.3. Hemoperitoneum volume unit odds of 1.43 corresponds with a 43% increase in the odds of splenectomy for each 100 mL increase in hemoperitoneum.

Clinical variables were not significantly different between the splenectomy and splenic salvage groups and were not included in multivariable analysis. For example, shock index at the time of follow-up was not significantly different between our splenectomy and splenic salvage samples (*p* = 0.77), with a median value < 1 (0.65 IQR: 0.62–0.67). The time between initial and follow-up CT trended lower (*p* = 0.11), and the transfusion requirement trended higher (*p* = 0.15), pointing to a higher degree of clinical severity in the splenectomy group.

The 12% incidence of splenectomy in our NOM cohort is comparable to the 8–11% incidence described in several prior works (10–12). Grade V injuries are typically managed with urgent splenectomy at our institution, and there were no such injuries in our follow-up CT cohort. We find that once hemodynamically stable patients are selected for an initial trial of NOM using admission CT-based AAST grading, that the resulting narrow distribution of grade II-IV injuries has limited clinical utility for predicting splenectomy. Our model incorporating hemoperitoneum volumes and presence of pseudoaneurysm on follow-up CT had significantly higher AUC than AAST grades for this outcome (0.91 vs. 0.59, *p* = 0.025).

Radiation concerns remain an important consideration in weighing the risks and benefits of follow-up CT imaging for young BSI patients selected for NOM (25). Evidence from Japanese atomic bomb survivors indicates that doses of ionizing radiation >100 mSv can increase the risk of cancer (46, 47) and this becomes relevant when multiple multi-phase CT examinations are performed over a short period (48). In our sample, the mean combined effective dose was 37.4 mSv (95% CI: 32.4–42.4). No patient received more than 100 mSv of radiation and the minimum age at exam was 24 years.

The immunological consequences of splenectomy should also be considered when implementing an NOM protocol that incorporates follow-up CT. In our splenectomy sample, 3 patients had findings consistent with delayed splenic rupture on follow-up CT, and in the other three, splenectomy was offered following CT due in part to a worsening constellation of imaging findings suggesting impending rupture. The potential benefit of reducing morbidity and mortality using follow-up CT should be weighed against the risk of future OPSI.

Overall, our findings support a role for follow-up CT in patients selected for a trial of non-operative management for BSI. The combination of pseudoaneurysm and hemoperitoneum volume on the follow-up CT study predicted splenectomy with high accuracy.

Our study had several limitations. Even though model AUC was high, our small sample size contributed to wide confidence intervals for the odds ratios of individual predictors. Clinical variables play a critical role in decision-making and lack of significant differences in clinical covariates were also likely related to the sample size. Our CT volumetry-based method provides a more objective and granular quantitative assessment of risk, however quantitative imaging is not currently feasible for BSI patients at the point of care, and this remains a research tool. Mean segmentation times for hemoperitoneum alone exceed 20 min (31). Semi-automated region-growing based algorithms with regulatory approval can potentially be used to obtain measurements of individual features of organ injury or hemorrhage (32, 33, 49), however the effort and expertise required precludes widespread acceptance, especially when multiple features are to be measured (50). Several proof-of-concept deep learning-based computer vision algorithms have been reported for rapid quantification of hemoperitoneum and splenic injury burden (31, 51). Steps required before such computer vision technology is ready for clinical adoption include translation into containerized software tools, validation in simulated deployment studies, regulatory approval, and clinical validation through multi-center studies.

## Conclusion

Follow-up CT assists in the decision to pursue splenectomy in BSI patients selected for an initial trial of NOM by providing imaging information that supplements the patient’s clinical status. New or persistent pseudoaneurysm and large hemoperitoneum volumes at follow-up are independent predictors of splenectomy. Each 100 mL increase in hemoperitoneum increases the odds of splenectomy by 43%.

## Figures and Tables

**TABLE 1:** Demographic and clinical characteristics.

Covariate	Total cohort (*n* = 51)	Splenectomy (*n* = 6)	Splenic salvage (*n* = 45)	*p*
Age (median[IQR])	40 (27–61)	63 (31–67)	40 (27–59)	0.56
**Gender (*n*[%])**				
Male	30 (59)	3 (50)	18 (40)	0.68
Female	21 (41)	3 (50)	21(38)	
Follow-up SBP^a^ (mean[*SD*])	133.9 (23.4)	141.0 (22.0)	133.0 (23.6)	0.41
Follow-up HR^b^ (median[IQR])	84 (74–101)	83 (77–97)	84 (73–100)	0.73
Follow-up SI (median[IQR])	0.65 (0.52–0.78)	0.65 (0.62–0.67)	0.66 (0.50–0.79)	0.77
AE (*n*[%])	10 (20)	1 (17)	9 (20)	1
Pseudoaneurysm (*n*[%])	13 (25)	5 (83)	8 (18)	**0.003**
Time between CTs^c^ (h) (median[IQR])	2.0 (1.3, 2.6)	1.4 (1.0, 2.0)	2.0 (1.4, 2.6)	0.11
Change in Hgb (mean[*SD*])	−2.2 (1.9)	−1.2 (1.5)	−2.3 (2.0)	0.11
PRBCs transfused (mean[*SD*])	1.7 (2.8)	3.0 (4.1)	1.5 (2.6)	0.15
AAST grade (*n*[%])^d^				0.63
1	2 (3.9)	0 (0.0)	2 (4.4)	
2	31 (61.0)	3 (50.0)	28 (62.2)	
3	12 (23.5)	2 (33.3)	10 (22.2)	
4	6 (11.8)	1 (16.7)	5 (11.1)	

Two-tailed *p* <0.05 indicate statistical significance and are bolded.

SD, Standard Deviation; IQR, Interquartile range; SBP, Systolic blood pressure; HR, Heart rate; SI, shock index; AE, Angioembolization; PRBCs, units packed red blood cells transfused.

a Systolic blood pressure at time of follow-up CT scan.

b Heart rate at time of follow-up CT scan.

c Time between initial CT and follow up CT.

d There were no conservatively managed grade V BSIs in our sample.

**TABLE 2:** Volumetric measurements

Covariate	Total cohort (*n* = 60)	Splenectomy (*n* = 7)	Splenic salvage (*n* = 53)	*p*
Final HPvol (mL) (median[IQR])	15.2 (2.1 to 89.3)	187.4 (96.9 to 550.7)	12.1 (0.9 to 53.4)	**0.003**
Final LACvol (mL) (median[IQR])	0.5 (0.0 to 4.1)	1.2 (0.2 to 10.1)	0.4 (0.0 to 3.2)	0.49
Final SCHvol (mL) (median[IQR])	0 (0 to 0)	5.0 (0 to 11.7)	0 (0 to 0)	**0.001**
∆ in HPvol/day (mL/day) (median[IQR])	0 (−8.1 to −8.1)	4.2 (−19.8 to 35.9)	0 (−5.6 to 6.7)	0.98
∆ in LACvol/day (mL/day) (median[IQR])	−0.1 (−0.9 to 0.1)	0 (−0.5 to 1.0)	−0.1 (−1.1 to 0.0)	0.52
∆ in SCHvol/day (mL/day) (median[IQR])	0 (0 to 0)	3.0 (0 to 9.9)	0 (0 to 0)	**0.09**

Two-tailed *p* < 0.1 are bolded and met inclusion criterion for model selection.

HPvol, Hemoperitoneum volume; LACvol, Laceration volume; SCHvol, Subcapsular hematoma volume.

**TABLE 3:** Multivariable model of the relationship between predictor variables and splenectomy.

Predictor	OR	95% CI	p
Final hemoperitoneum volume per 100 mL^a^	1.43	0.99–2.06	0.055
Pseudoaneurysm Yes vs. No	160.3	0.91–28315.3	0.054

OR, odds ratio.

a Each 100 mL increase in final hemoperitoneum volume corresponds with a 43% increase in odds of splenectomy.

## Data Availability

The raw data supporting the conclusions of this article will be made available by the authors, without undue reservation.

## References

[1] Tom WW, Howells GA, Bree RL, Schwab R, Lucas RJ. A nonoperative approach to the adult ruptured spleen sustained from blunt trauma. Am Surg (1985) 51:367–71. 4014879

[2] Stivelman RL, Glaubitz JP, Crampton RS. Laceration of the spleen due to nonpenetrating trauma: one hundred cases. Am J Surg (1963) 106:888–91. doi: 10.1016/0002-9610(63)90151-X 14099463

[3] Umlas SL, Cronan JJ. Splenic trauma: can CT grading systems enable prediction of successful nonsurgical treatment? Radiology (1991) 178:481–7. doi: 10.1148/radiology.178.2.1987612 1987612

[4] Lynch AM, Kapila R. Overwhelming postsplenectomy infection. Infect Dis Clin North Am (1996) 10:693–707. doi: 10.1016/S0891-5520(05)70322-6 8958164

[5] Holdsworth R, Cuschieri A, Irving A. Postsplenectomy sepsis and its mortality rate: actual versus perceived risks. Br J Surg (1991) 78:1031–8. doi: 10.1002/bjs.1800780904 1933181

[6] Teuben M, Spijkerman R, Teuber H, Pfeifer R, Pape H-C, Kramer W, Splenic injury severity, not admission hemodynamics, predicts need for surgery in pediatric blunt splenic trauma. Patient Saf Surg (2020) 14:1. doi: 10.1186/s13037-019-0218-0 31911819 PMC6942310

[7] Green JB, Shackford SR, Sise MJ, Fridlund P. Late septic complications in adults following splenectomy for trauma: a prospective analysis in 144 patients. J Trauma (1986) 26:999–1004. doi: 10.1097/00005373-198611000-00007 3783791

[8] Renz BM, Feliciano DV. Unnecessary laparotomies for trauma: a prospective study of morbidity. J Trauma (1995) 38:350–6. doi: 10.1097/00005373-199503000-00007 7897713

[9] Zarzaur BL, Kozar R, Myers JG, Claridge JA, Scalea TM, Neideen TA, The splenic injury outcomes trial: an American Association for the Surgery of Trauma multi-institutional study. J Trauma Acute Care Surg (2015) 79:335–42. doi: 10.1097/TA.0000000000000782 26307863

[10] Bee TK, Croce MA, Miller PR, Pritchard FE, Fabian TC. Failures of splenic nonoperative management: is the glass half empty or half full? J Trauma (2001) 50:230–6. doi: 10.1097/00005373-200102000-00007 11242286

[11] Peitzman AB, Heil B, Rivera L, Federle MB, Harbrecht BG, Clancy KD, Blunt splenic injury in adults: Multi-institutional Study of the Eastern Association for the Surgery of Trauma. J Trauma (2000) 49:177–87; discussion 187–179. doi: 10.1097/00005373-200008000-00002 10963527

[12] Nix JA, Costanza M, Daley BJ, Powell MA, Enderson BL. Outcome of the current management of splenic injuries. J Trauma (2001) 50:835–42. doi: 10.1097/00005373-200105000-00010 11371838

[13] Peitzman AB, Harbrecht BG, Rivera L, Heil B. Failure of observation of blunt splenic injury in adults: variability in practice and adverse consequences. J Am Coll Surg (2005) 201:179–87. doi: 10.1016/j.jamcollsurg.2005.03.037 16038813

[14] Leeper WR, Leeper TJ, Ouellette D, Moffat B, Sivakumaran T, Charyk-Stewart T, Delayed hemorrhagic complications in the nonoperative management of blunt splenic trauma: early screening leads to a decrease in failure rate. J Trauma Acute Care Surg (2014) 76:1349–53. doi: 10.1097/TA.0000000000000228 24854299

[15] Lawson DE, Jacobson JA, Spizarny DL, Pranikoff T. Splenic trauma: value of follow-up CT. Radiology (1995) 194:97–100. doi: 10.1148/radiology.194.1.7997589 7997589

[16] Federle MP. Splenic trauma: is follow-up CT of value? Radiology (1995) 194:23–4. doi: 10.1148/radiology.194.1.7997559 7997559

[17] Weinberg JA, Magnotti LJ, Croce MA, Edwards NM, Fabian TC. The utility of serial computed tomography imaging of blunt splenic injury: still worth a second look? J Trauma Acute Care Surg (2007) 62:1143–8. doi: 10.1097/TA.0b013e318047b7c2 17495714

[18] Furlan A, Tublin ME, Rees MA, Nicholas DH, Sperry JL, Alarcon LH. Delayed splenic vascular injury after nonoperative management of blunt splenic trauma. J Surg Res (2017) 211:87–94. doi: 10.1016/j.jss.2016.11.062 28501136

[19] Haan JM, Bochicchio GV, Kramer N, Scalea TM. Nonoperative management of blunt splenic injury: a 5-year experience. J Trauma Acute Care Surg (2005) 58:492–8. doi: 10.1097/01.TA.0000154575.49388.74 15761342

[20] Harbrecht BG. Is anything new in adult blunt splenic trauma? Am J Surg (2005) 190:273–8. doi: 10.1016/j.amjsurg.2005.05.026 16023445

[21] Thaemert BC, Cogbill TH, Lambert PJ. Nonoperative management of splenic injury: are follow-up computed tomographic scans of any value? J Trauma Acute Care Surg (1997) 43:748–51. doi: 10.1097/00005373-199711000-00003 9390484

[22] Zarzaur BL, Rozycki GS. An update on nonoperative management of the spleen in adults. Trauma Surg Acute Care Open (2017) 2:e000075. doi: 10.1136/tsaco-2017-000075 29766085 PMC5877897

[23] Bhangu A, Nepogodiev D, Lal N, Bowley DM. Meta-analysis of predictive factors and outcomes for failure of non-operative management of blunt splenic trauma. Injury (2012) 43:1337–46. doi: 10.1016/j.injury.2011.09.010 21999935

[24] Brillantino A, Iacobellis F, Robustelli U, Villamaina E, Maglione F, Colletti O, Non operative management of blunt splenic trauma: a prospective evaluation of a standardized treatment protocol. Eur J Trauma Emerg Surg (2016) 42:593–8. doi: 10.1007/s00068-015-0575-z 26416401

[25] Sinha S, Raja S, Lewis M. Recent changes in the management of blunt splenic injury: effect on splenic trauma patients and hospital implications. Ann R Coll Surg Engl (2008) 90:109–12. doi: 10.1308/003588408X242033 18325207 PMC2443302

[26] Odedra D, Mellnick V, Patlas M. A 2019 international survey to assess trends in follow-up imaging of blunt splenic trauma. Emerg Radiol (2020) 27:51–6. doi: 10.1007/s10140-019-01734-8 31691876

[27] Boscak AR, Shanmuganathan K, Mirvis SE, Fleiter TR, Miller LA, Sliker CW, Optimizing trauma multidetector CT protocol for blunt splenic injury: need for arterial and portal venous phase scans. Radiology (2013) 268:79–88. doi: 10.1148/radiol.13121370 23449955

[28] Uyeda JW, LeBedis CA, Penn DR, Soto JA, Anderson SW. Active hemorrhage and vascular injuries in splenic trauma: utility of the arterial phase in multidetector CT. Radiology (2014) 270:99–106. doi: 10.1148/radiol.13121242 24056401

[29] Melikian R, Goldberg S, Strife BJ, Halvorsen RA. Comparison of MDCT protocols in trauma patients with suspected splenic injury: superior results with protocol that includes arterial and portal venous phase imaging. Diagn Interv Radiol (2016) 22:395–9. doi: 10.5152/dir.2016.15232 27334296 PMC5019842

[30] Federle M, Jeffrey R Jr. Hemoperitoneum studied by computed tomography. Radiology (1983) 148:187–92. doi: 10.1148/radiology.148.1.6856833 6856833

[31] Dreizin D, Zhou Y, Fu S, Wang Y, Li G, Champ K, A multiscale deep learning method for quantitative visualization of traumatic hemoperitoneum at CT: assessment of feasibility and comparison with subjective categorical estimation. Radiology (2020) 2:e190220. doi: 10.1148/ryai.2020190220 33330848 PMC7706875

[32] Battey TW, Dreizin D, Bodanapally UK, Wnorowski A, Issa G, Iacco A, A comparison of segmented abdominopelvic fluid volumes with conventional CT signs of abdominal compartment syndrome in a trauma population. Abdom Radiol (2019) 44:2648–55. doi: 10.1007/s00261-019-02000-8 30953097

[33] Dreizin D, Bodanapally U, Boscak A, Tirada N, Issa G, Nascone JW, CT prediction model for major arterial injury after blunt pelvic ring disruption. Radiology (2018) 287:1061–9. doi: 10.1148/radiol.2018170997 29558295

[34] Dreizin D, Bodanapally U, Mascarenhas D, O’Toole RV, Tirada N, Issa G, Quantitative MDCT assessment of binder effects after pelvic ring disruptions using segmented pelvic haematoma volumes and multiplanar caliper measurements. Eur Radiol (2018) 28:3953–62. doi: 10.1007/s00330-018-5303-8 29536245

[35] Winer-Muram HT, Jennings SG, Tarver RD, Aisen AM, Tann M, Conces DJ, Volumetric growth rate of stage I lung cancer prior to treatment: serial CT scanning. Radiology (2002) 223:798–805. doi: 10.1148/radiol.2233011026 12034952

[36] Buckler AJ, Mulshine JL, Gottlieb R, Zhao B, Mozley PD, Schwartz L. The use of volumetric CT as an imaging biomarker in lung cancer. Acad radiol (2010) 17:100–6. doi: 10.1016/j.acra.2009.07.030 19969253

[37] Rowell SE, Biffl WL, Brasel K, Moore EE, Albrecht RA, DeMoya M, Western Trauma Association Critical Decisions in Trauma: management of adult blunt splenic trauma-−2016 updates. J Trauma Acute Care Surg (2017) 82:787–93. doi: 10.1097/TA.0000000000001323 27893644

[38] Duchesne JC, Simmons JD, Schmieg RE Jr, McSwain NE Jr, Bellows CF. Proximal splenic angioembolization does not improve outcomes in treating blunt splenic injuries compared with splenectomy: a cohort analysis. J Trauma Acute Care Surg (2008) 65:1346–53. doi: 10.1097/TA.0b013e31818c29ea 19077625

[39] Velmahos GC, Chan LS, Kamel E, Murray JA, Yassa N, Kahaku D, Nonoperative management of splenic injuries: have we gone too far? Arch Surg (2000) 135:674–81. doi: 10.1001/archsurg.135.6.674 10843363

[40] Fedorov A, Beichel R, Kalpathy-Cramer J, Finet J, Fillion-Robin J-C, Pujol S, 3D Slicer as an image computing platform for the Quantitative Imaging Network. Magn Reson Imaging (2012). 30:1323–41. doi: 10.1016/j.mri.2012.05.001 22770690 PMC3466397

[41] Dreizin D, Chen T, Liang Y, Zhou Y, Paes F, Wang Y, Added value of deep learning-based liver parenchymal CT volumetry for predicting major arterial injury after blunt hepatic trauma: a decision tree analysis. Abdom Radiol (2021) 46:2556–66. doi: 10.1007/s00261-020-02892-x PMC8205942 33469691

[42] Dreizin D, Zhou Y, Zhang Y, Tirada N, Yuille AL. Performance of a deep learning algorithm for automated segmentation and quantification of traumatic pelvic hematomas on CT. J Digit Imaging (2020) 33:243–51. doi: 10.1007/s10278-019-00207-1 31172331 PMC7064706

[43] Hanley JA, McNeil BJ. The meaning and use of the area under a receiver operating characteristic (ROC) curve. Radiology (1982) 143:29–36. doi: 10.1148/radiology.143.1.7063747 7063747

[44] Stassen NA, Bhullar I, Cheng JD, Crandall ML, Friese RS, Guillamondegui OD, Selective nonoperative management of blunt splenic injury: an Eastern Association for the Surgery of Trauma practice management guideline. J Trauma Acute Care Surg (2012) 73:S294–300. doi: 10.1097/TA.0b013e3182702afc 23114484

[45] Pachter HL, Guth AA, Hofstetter SR, Spencer FC. Changing patterns in the management of splenic trauma: the impact of nonoperative management. Ann Surg (1998) 227:708–717; discussion 717–709. doi: 10.1097/00000658-199805000-00011 9605662 PMC1191351

[46] Ali YF, Cucinotta FA, Ning-Ang L, Zhou G. Cancer risk of low dose ionizing radiation. Front Phys (2020) 8:234. doi: 10.3389/fphy.2020.00234

[47] Health risks from exposure to low levels of ionizing radiation: BEIR VII phase 2: National Research Council (2006). 25077203

[48] Lin EC. Radiation Risk From Medical Imaging Mayo Clinic Proceedings: Elsevier (2010). p. 1142–6. doi: 10.4065/mcp.2010.0260 10.4065/mcp.2010.0260 PMC2996147 21123642

[49] Dreizin D, Bodanapally UK, Neerchal N, Tirada N, Patlas M, Herskovits E. Volumetric analysis of pelvic hematomas after blunt trauma using semi-automated seeded region growing segmentation: a method validation study. Abdom Radiol (2016) 41:2203–8. doi: 10.1007/s00261-016-0822-8 27349420

[50] Borror W, Gaski GE, Steenburg S. Abdominopelvic bleed rate on admission CT correlates with mortality and transfusion needs in the setting of blunt pelvic fractures: a single institution pilot study. Emerg Radiol (2019) 26:37–44. doi: 10.1007/s10140-018-1646-3 30259226

[51] Zhou Y, Dreizin D, Wang Y, Liu F, Shen W, Yuille AL. External attention assisted multi-phase splenic vascular injury segmentation with limited data. IEEE Trans Med Imaging (2021) 41:1346–57. doi: 10.1109/TMI.2021.3139637 PMC9167782 34968179

